# Strengths and opportunities in research into extracellular matrix ageing: A consultation with the ECMage research community

**DOI:** 10.1002/bies.202300223

**Published:** 2024-03-24

**Authors:** Matthew J. Dalby, Vanja Pekovic‐Vaughan, Daryl P. Shanley, Joe Swift, Lisa J. White, Elizabeth G. Canty‐Laird

**Affiliations:** ^1^ Centre for the Cellular Microenvironment Institute of Molecular Cell and Systems Biology College of Medical Veterinary and Life Sciences University of Glasgow Glasgow UK; ^2^ Department of Musculoskeletal and Ageing Science Institute of Life Course and Medical Sciences Faculty of Health and Life Sciences William Henry Duncan Building University of Liverpool Liverpool UK; ^3^ Campus for Ageing and Vitality Biosciences Institute Newcastle University Newcastle‐upon‐Tyne UK; ^4^ Wellcome Centre for Cell‐Matrix Research University of Manchester Manchester UK; ^5^ School of Pharmacy and Biodiscovery Institute University of Nottingham University Park Nottingham UK; ^6^ The Medical Research Council Versus Arthritis Centre for Integrated Research into Musculoskeletal Ageing (CIMA) University of Liverpool Liverpool UK

**Keywords:** ageing, biophysics, chronobiology, extracellular matrix, model systems

## Abstract

Ageing causes progressive decline in metabolic, behavioural, and physiological functions, leading to a reduced health span. The extracellular matrix (ECM) is the three‐dimensional network of macromolecules that provides our tissues with structure and biomechanical resilience. Imbalance between damage and repair/regeneration causes the ECM to undergo structural deterioration with age, contributing to age‐associated pathology. The ECM ‘Ageing Across the Life Course’ interdisciplinary research network (ECMage) was established to bring together researchers in the United Kingdom, and internationally, working on the emerging field of ECM ageing. Here we report on a consultation at a joint meeting of ECMage and the Medical Research Council / Versus Arthritis Centre for Integrated Research into Musculoskeletal Ageing, held in January 2023, in which delegates analysed the key questions and research opportunities in the field of ECM ageing. We examine fundamental biological questions, enabling technologies, systems of study and emerging in vitro and in silico models, alongside consideration of the broader challenges facing the field.

## INTRODUCTION

Advances in science, medicine, and living conditions mean that people are living longer. Whilst a longer life brings exciting opportunities, ageing is too often associated with ill health and frailty.^[^ ^1^ ^]^ The ageing demographic brings with it a steep increase in the public burden of health and social care as well as financial challenges. Across the United Kingdom (UK), and globally, there are inequalities in the lived experience of ageing, with social demographics, life course experiences, environmental, and genetic factors influencing stark disparities between chronological and biological age in different populations.

The process of ageing has been systematically characterised from a biological perspective.^[^ ^2^ ^]^ Recently, there has been growing interest in the role in ageing of the extracellular matrix (ECM), a three‐dimensional network of molecules which not only provides resilience to all tissues in the body, but also controls key biophysical and biochemical signals to support vital cell functions.^[^ ^3^ ^]^ The ECM makes up the bulk of most tissues, acting as the primary determinant of biomechanical properties. For example, in the bone, the ECM is mineralised providing essential strength, whilst in the lung, it provides elasticity necessary for normal function. The ECM in each organ is distinct and dynamic,^[^ ^4^ ^]^ responding to various external factors such as day/night cycles,^[^ ^5^ ^]^ highlighting its intrinsic malleability. With age, the ECM deteriorates through a process influenced by an imbalance between its production and turnover, chemical changes to molecules, as well as changes to cell‐ECM interactions. Importantly, ECM deterioration with age directly influences key functions of cells and tissues in the body leading to altered mechanical properties,^[^ ^6^ ^]^ abnormal cell signalling, and loss of vital functions. As a result, changes to ECM underpin many age‐associated pathologies, such as a loss of tissue elasticity (e.g., in lung, blood vessels, skin), poor tissue repair in response to injury (e.g., chronic wounds, tendinopathies), tissue scarring (fibrosis), risk of bone fracture, joint deterioration,^[^ ^7^ ^]^ and cancer.^[^ ^8^ ^]^

**FIGURE 1:**
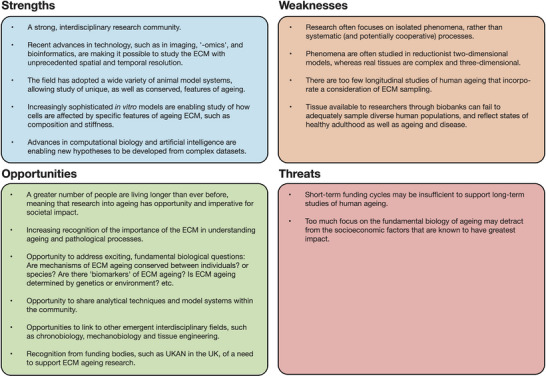
Identification of strengths, weaknesses, opportunities, and threats associated with research into extracellular matrix (ECM) ageing.

The UK Ageing Networks (UKAN, https://www.ukanet.org.uk) were established in 2022 with UK Research and Innovation (UKRI) funding to coordinate research that addresses these challenges. Within this structure, the ECM ‘Ageing Across the Life Course’ interdisciplinary research network (ECMage) was set up to promote understanding of ageing in the ECM and to identify suitable model systems and potential interventions to improve health and well‐being. ECMage brings together a diverse membership in terms of career stage and expertise in key aspects of ageing, matrix biology, chronobiology, artificial intelligence (AI), computational modelling, and tissue engineering. Establishing understanding of ECM ageing across the life course poses particular challenges. For example, there is a need to establish longitudinal studies in humans, and for the development of new model systems — in vivo, in vitro, and in silico. In order to assess the current capacity of the UK research community to address these challenges, ECMage organised a consultation event at a joint meeting with the Medical Research Council / Versus Arthritis Centre for Integrated Research into Musculoskeletal Ageing (CIMA) Meeting, held in York (UK) on 27^th^ January 2023. Delegates were asked to propose aspirational goals for the ageing matrix field, to compare these to the current state‐of‐the‐art, and to identify opportunities for new research and collaboration. Participants were invited to join working groups exploring different aspects underpinning the ECMage Network. Individual groups were asked to identify and critically appraise the research landscape within their respective topic areas. This report summarises key themes that were raised and recorded at the consultation (Figure 1).

## MECHANISMS AND INTERVENTIONS

The consultation recognised a number of open and fundamental biological questions within the ECM‐ageing field. These included questions such as: the extent to which the ageing process is conserved between individuals; whether there are universal signatures or ‘biomarkers’ of ageing across different organs or species; the importance of genetic, environmental, and behavioural, variation in modulating the ageing process (e.g., sun exposure, occupational history, dietary habits, alcohol intake); and, whether ageing follows a continuous and life‐long progression (perhaps akin to the accrual of photo‐induced damage in exposed skin), or whether it can be ‘accelerated’ following certain events (e.g., injury or menopause), or within discrete periods. More specifically to the ECM, a fundamental understanding of how specific cells deposit and remodel their surrounding tissue environments was considered necessary: how are processes of ECM remodelling and regeneration affected by ageing? Are age‐associated tissue defects established by changes in intracellular processes (e.g., through senescence or DNA damage pathways) or extracellular (e.g., through oxidative damage, hormone imbalance, ECM factors)? This can be posited as a question of whether key signalling processes that underpin the ECM ageing axis occur inside‐out, or outside‐in, from the perspective of the cell — or some reciprocal combination of both. It was discussed whether and how the ECM might be affected by pharmaceutical and life‐style interventions such as exercise, diet, and medicines. Whilst it is known that models of accelerated ageing show a dysfunctional ECM and a ‘younger ECM’ can rejuvenate old cells, limited research has been done to show that ECM‐based interventions can influence the rate of intrinsic aging.^[^ ^9^ ^]^ Thus, there is critical need to expand our knowledge in this area.

A growing interest in the study of extracellular vesicles (EVs) was also discussed:^[^ ^10^ ^]^ could these represent a signalling reservoir of ECM molecules that is disrupted in ageing? Are EVs maintained in tissues? Do EVs bind to specific ECM molecules, or in a tissue‐specific manner? And could EVs be a target for diagnostic or therapeutic strategies? Participants expressed questions relating to the study of mechanobiology and ECM‐cell communication or ‘mechanotransduction’:^[^ ^6^ ^]^ What forces do specific cells and organs experience? What forces can cells ‘feel’ and respond to? How are these processes affected by ageing, particularly as many tissues stiffen during ageing? What factors link the structure, function and mechanical properties of tissues? Furthermore, we do not know whether targeting either ECM components or cell/ECM interactions in the ageing context might alleviate tissue dysfunction. It is still unknown which ECM‐producing cell types in various organ systems may be responsible for specific age‐dependent changes.

A major challenge for researchers is that current models tend to be designed on reductionist principles; should we be considering more complex three‐dimensional or multi‐component in vitro models, considering both cell‐cell and cell‐matrix interactions? Furthermore, the current literature tends to focus on signalling pathways in isolation (e.g., adhesion complexes, ion channels, transcription factor translocation) — to what extent might these processes be cooperative? Emerging research has shown that the ECM is a target of the daily changes governed by the universal time‐keeping mechanism in the body, the circadian clock,^[^ ^5^ ^]^ highlighting the need to understand how this temporal regulation of structure, function, and biomechanics are affected by ageing.

It was recognised that the challenges discussed above were in many cases being compounded by common constraints, including limited access to expertise, technologies, and models, but that these might be alleviated by sharing of improved analytical techniques and model systems of study.

## ADVANCED TECHNOLOGIES

A holistic examination of ageing must consider processes that occur in short timescales, through to whole lifetimes, and over length scales spanning molecular, cellular, and tissue‐sized dimensions. Moreover, strategies must be developed to integrate and unify understanding from these multiple means of study. Participants discussed emerging methods such as X‐ray scattering to study the organisation of ECM molecules in tissues, and the need to integrate this data with a better understanding of tissue composition. A wider range of imaging methods (e.g. super‐resolution microscopy, light sheet microscopy, imaging mass cytometry, real‐time bioluminescence imaging) are also needed that can study molecular and cellular processes within ‘thick’ sections of material that are more reflective of living tissues.

Mass spectrometry proteomics can inform on how the composition of tissues changes during ageing. Advances in instrumentation are making it possible to characterise the proteome with greater spatial and temporal resolution. Questions were identified regarding how protein metabolism — synthesis, degradation and turnover — is affected by ageing in different cells and tissues, and on the roles of post‐translational modifications (PTMs). It was commented that establishing a ‘multi‐omic atlas’ of ECM ageing could be a powerful tool for hypothesis generation. There was recognition of the challenge to ensure that very complex, multi‐dimensional datasets can be stored and searched, that they remain current and accessible, and crucially, that they are fully utilised. However, many of these issues are being addressed through adoption of community projects such as MatrisomeDB.^[^ ^4^ ^]^

## POPULATION‐BASED AND IN VIVO SYSTEMS OF STUDY

Participants recognised a need for more comprehensive longitudinal studies in humans. As the factors affecting old age may change throughout the life course, it was considered insufficient to study the ageing processes only in the elderly. Given that the course of ageing varies greatly between individuals depending on their genetic makeup and environmental exposures, longitudinal studies (i.e., studying the same individuals over multiple time points) were considered preferable to studies reliant on averaging over populations. It was noted that longitudinal studies are challenging to set up and maintain as they are dependent on the long‐term commitment and vision of participants, researchers, and funders alike. Participants also discussed the challenges of working with biobanks: there is a need to adequately sample diverse human populations, and this should include tissues from young, middle‐aged and healthy donors. There are also important ethical issues in collecting and retaining sufficient meta‐data on patient samples in order to establish a holistic understanding of age‐contributing risk factors.

A range of experimental in vivo models of ageing were considered, including invertebrate models, rodents and larger mammals.^[^ ^11^ ^]^ There remain questions over the extent to which the features of ECM ageing are conserved between species. For example, short lived species such as killifish may have a different extent of cell‐independent ECM modification (e.g., glycation, carbamylation) when compared to longer lived species and humans. Importantly, there is no universal experimental model of ageing, and different models may be appropriate to studies of different aspects of ageing, processes, and tissues/organs. A challenge for researchers working with animal models is often to motivate (for example, to funders) a relevance to the understanding of human ageing, and demonstrate a pathway to translation into diagnostic or therapeutic technology.

## TISSUE ENGINEERING AND NOVEL IN VITRO MODELS

Recent advances in tissue engineering and the fabrication of sophisticated in vitro model systems have offered alternative — or often complimentary — means to study aspects of ECM‐ageing in humans and animals.^[^ ^12^ ^]^ An advantage of in vitro over in vivo models is that they can allow control of individual parameters within a system, for example, it would be feasible to decouple the roles of biochemical and mechanical effects in influencing a particular cellular behaviour. Nonetheless, there remains a challenge to adequately balance a reductionist model with one that better replicates the complexity of a tissue, but has too many confounding variables.

Participants considered that there remains a lack of suitable in vitro models to study ECM ageing, for example, to aid in the understanding of oxidative and glycaemic stress factors. More broadly, whilst the use of biomaterials for development of in vitro or *ex vivo* cellular scaffolds (e.g., hydrogels based on synthetic, or non‐synthetic ECM‐derived components, or decellularized tissues) has potential to transform ECM ageing research, the capacity to fabricate a completely functional synthetic ECM remains a challenge for the bioengineering field.^[^ ^12^ ^]^ New technologies are also required to produce thicker materials, and to produce more complex three‐dimensional tissue structures and topologies, such as to allow functional vascularisation. Within the context of tissue engineering, the incorporation of stems cells into model and therapeutic systems was discussed. Challenges remain on how to safely and efficiently direct stem cell behaviours, such as differentiation, and how to adequately scale up stem cell populations for medical applications.

## COMPUTATIONAL MODELS

The importance of interdisciplinary approaches combining computer science, mathematics and bioinformatics was recognised as being vital to constructing a complete understanding of ECM ageing. These disciplines will be necessary in processing, integrating and interpreting multi‐dimensional datasets, enabling a comprehensive understanding of the roles of genetic, transcriptomic, proteomic, post‐translational, and metabolomic regulation in the ECM ageing process. Computational models combined with systems biology approaches will help us to understand cell‐ECM and cell‐biomaterial interactions from micro to macro level, thus complimenting the *in vitr* *o* and in vivo experimental models.^[^ ^13^ ^]^ Likewise, mathematical models will help us to predict long term and population‐level outcomes within timeframes shorter than the human lifespan, whilst systems pharmacology modelling approaches will enable us predict novel matrix‐based anti‐ageing interventions. Finally, participants discussed how machine learning and AI could affect ECM ageing research. This is a fast‐emerging and potentially revolutionary technology, but the extent of its impact on ageing research is difficult to predict. Nonetheless, AI has the potential to identify patterns and connections within ECM datasets that we have not yet begun to appreciate.

## BIG QUESTIONS FOR SCIENCE AND SOCIETY

The challenges facing research into ECM ageing raised in this consultation require an inter‐disciplinary approach, combining aspects of molecular and cellular biology, physiology/medicine, structural biology/biophysics, chronobiology, bioengineering, and computational/AI modelling, among others. However, the scientific questions raised in the discussion above must also be viewed in a wider societal context, requiring wider input from our stakeholders. Big questions for us all to consider include: how should we define and measure age? Should it be strictly defined by the passage of time, or can we surrogate it with a combination of biological processes? (Acquisition of pathology? Abrogation of cellular processes such as metabolism? Or damage accumulation in the ECM?) Should we frame the study of ageing as a progressive accruement of tissue pathology, or the progression of a natural physiological process? Should the measure of success be in improved quality of life, or longevity, or both? Finally, given that we already know many of the risk factors associated with unhealthy ageing (e.g., smoking, malnutrition, weight gain, a sedentary lifestyle, exposure to pollution, social isolation/loneliness, drug/alcohol abuse), and that effective interventions are socioeconomic in nature, how should our resources be targeted for maximum impact?

To maximise the potential benefits to society from research in fundamental science, as well as from bioindustry and biomedicine, it is critical that the ECM is considered in both experimental and clinical studies of ageing. Understanding of ECM ageing in human populations will provide unique opportunities to design improved therapies and interventions, positively influencing tissue resilience and increasing health span. By working together, sharing models, technologies, knowledge, and expertise, we will be better equipped to understand the complex intersections between intrinsic ageing processes, ECM regulation and the extrinsic factors that influence their crosstalk. This will improve our ability to meet the challenges of increasing the quality of life of older people.

## CONFLICT OF INTEREST STATEMENT

The authors declare no conflicts of interest.

## Data Availability

The data that support the findings of this study are available from the upon reasonable request.
